# Editorial: New insights into cancer predisposition syndromes in pediatric hematology-oncology

**DOI:** 10.3389/fonc.2025.1580671

**Published:** 2025-03-11

**Authors:** Roberto Carta, Davide Leardini, Angela Mastronuzzi

**Affiliations:** ^1^ Department of Hematology and Oncology, Cell and Gene Therapy, Bambino Gesù Children’s Hospital [Istituto di Ricovero e Cura a Carattere Scientifico (IRCCS)], Rome, Italy; ^2^ Pediatric Hematology and Oncology, Istituto di Ricovero e Cura a Carattere Scientifico (IRCCS) Azienda Ospedaliero-Universitaria di Bologna, Bologna, Italy

**Keywords:** cancer, predisposition, syndromes, pediatric, oncology, hematology, genetic, children

Hereditary predisposition is a relevant cause of hematologic and solid cancer in children and adolescents. It has been estimated that at least 10% of all pediatric tumors are due to germline mutations in cancer predisposition genes, and recent evidence suggests that this number may be higher (1, 2). In recent years, advances in molecular biology and genetic technologies, such as large-scale genome sequencing analysis, have improved the biological and molecular understanding of Cancer Predisposition Syndromes (CPS) (3). However, many gray areas remain, such as the interpretation of the role of an identified genetic variant. Indeed, one of the most significant burdens is represented by the detection of Variants of Uncertain Significance (VUSs), with their uncertain role and impact on the pathogenesis of neoplasms.

For individuals who have already been diagnosed with cancer, knowing that a specific mutation is present can help choose targeted and effective treatment. In addition, the detection of a genetic variant in the patient may be important not only for the organization of follow-up strategies but also for the adoption of possible surveillance strategies in family members.

The aim of the Research Topic “*New Insights into Cancer Predisposition Syndromes in Pediatric Hematology-Oncology*”, which includes seven articles, is to highlight the most recent advances in epidemiological, clinical, biological, molecular, and therapeutic aspects of CPS in pediatric and adolescent populations and to provide new insights that may be useful to further improve the diagnosis, treatment, and follow-up of CPS.

Vinci et al. presented five instructive cases of GATA2-related diseases, offering a clinically valuable analysis of current knowledge and treatment approaches presented with useful learning points. GATA2 is an emerging gene in pediatric hematology with a strong association with malignancies (4, 5). The authors highlighted the expanding phenotype of GATA2 deficiency, which extends beyond hematologic involvement to include immunological and pulmonary manifestations. The authors appropriately emphasized the need for heightened awareness of this condition among hemato-oncology specialists to avoid delays in diagnosis. Among the unresolved questions, we particularly highlight the role of preemptive allogeneic transplantation—an approach with curative potential but significant risks. Importantly, patient perception should be a key factor in decision-making.

Di Benedetto et al. provided a valuable retrospective analysis of 23 pediatric and adolescent patients with RET-mutant medullary thyroid carcinoma, highlighting genotype-phenotype correlations and disease outcomes. Their findings reinforced the critical role of early detection through genetic screening and timely intervention. Notably, the authors emphasized the impact of RET mutations on prognosis, with M918T confirming its aggressive course. The study underscores the importance of monitoring first-degree relatives to optimize preventive strategies. While limited by its retrospective nature, this work advances our understanding of hereditary medullary thyroid carcinoma and informs clinical decision-making regarding the timing and extent of surgical management.

Rees et al. conducted exome sequencing of 160 medulloblastoma survivors, focusing on rare variants in 239 cancer susceptibility genes (CSGs). They identified pathogenic/likely pathogenic (P/LP) variants in CSGs in 12.5% of pediatric medulloblastoma survivors, compared to 5% of controls. Notably, ELP1 and SUFU were among the most enriched CSGs. The study also identified two novel genes, CHEK2 and AGL, that had not previously been linked to medulloblastoma. These findings highlight the high prevalence of P/LP variants in CSGs in medulloblastoma survivors and have important implications for genetic testing to guide risk assessment and personalized treatment strategies.

Fabozzi et al. in their article described a rare case of a 5-year-old girl carrying a VUS in the *Succinate Dehydrogenase Complex Subunit C* (*SHDC*) gene who developed a therapy-related acute myeloid leukemia as a second neoplasm after being treated for high-risk neuroblastoma. Their case report highlights the importance of performing genetic testing in patients who develop a second malignancy to identify both constitutional and somatic mutations. This case report also highlights the importance of functional studies to further investigate the potential impact and causative role on tumor susceptibility of the genetic variant identified.

Ling et al. in their paper described a rare case of paraneoplastic juvenile dermatomyositis (JDM) and Hodgkin’s lymphoma in an adolescent female patient. The authors emphasized the uniqueness of the case described and highlighted the differences in cancer risk compared to patients with adult-onset dermatomyositis. They demonstrated the importance of a detailed physical examination, which led them to detect two distinct and rarely related clinical entities and how treating the underlying neoplasm can also improve the associated paraneoplastic condition. The authors emphasized the need for tumor screening in patients with JDM and unusual clinical findings and for careful long-term follow-up even in cases in which an underlying malignancy is not detected when JDM is diagnosed.

Mak et al. reported on three pediatric cases of medulloblastoma with subsequent development of therapy-related myeloid neoplasms as a potential complication of treatment of the primary tumor. The authors emphasized the importance of discovering a cancer predisposition syndrome that may play a causal role, have significant prognostic implications, and predict treatment response. They also highlighted the importance of surveillance for hematological abnormalities in long-term medulloblastoma survivors.

Greene et al. described the importance of performing molecular testing on somatic samples from pediatric CNS neoplasms using a multiplexed targeted next-generation sequencing panel validated to detect genetic alterations in various cancer-related genes. They described how obtaining information about the somatic molecular sequencing of the tumor can help select a subpopulation of patients who deserve to be considered for germline analysis. Confirmation of a germline variant for some patients can be important in clarifying the diagnosis, because informing a patient about their cancer risk can lead to life-saving surveillance and risk reduction interventions for themselves and their family members, and can sometimes directly impact their treatment plan.

Indeed, the application of molecular sequencing methods still has some hurdles to overcome. As mentioned by Greene et al., the application of somatic sequencing in tumor tissue samples at diagnosis or relapse of the disease may provide insights and help to select a subpopulation of patients in whom germline testing should be recommended. However, the criteria for such selection have not been universally defined and may vary from laboratory to laboratory. A useful parameter for recommending germline variant detection from somatic samples is variant allele frequency. However, this may be influenced by other variables such as tumor tissue heterogeneity and copy number alterations. Selecting patients for germline variant detection from somatic data can be time-consuming, depending on the laboratory, and sometimes the results may come too late, once the patient’s treatment has already been performed, not allowing to avoid potential treatment-related complications. For this reason, some authors have suggested performing paired somatic and germline testing at the time of primary tumor diagnosis. While this proposal is certainly advantageous and provides accurate pre-treatment information, it may be too expensive for centers with more limited financial resources.

It is important to underline that once genetic information has been acquired, an explanation of the results needs to be provided as part of structured genetic counseling.

To conclude, CPS remains a challenging issue in pediatric hematology and oncology, and much effort is still needed to better understand the impact of molecular sequencing on the clinical management and outcomes of patients and the psychological aspects of all family members.
